# The Epidemiology of Delirium: Challenges and Opportunities for Population Studies

**DOI:** 10.1016/j.jagp.2013.04.007

**Published:** 2013-12

**Authors:** Daniel H.J. Davis, Stefan H. Kreisel, Graciela Muniz Terrera, Andrew J. Hall, Alessandro Morandi, Malaz Boustani, Karin J. Neufeld, Hochang Benjamin Lee, Alasdair M.J. MacLullich, Carol Brayne

**Affiliations:** a Department of Public Health and Primary Care, University of Cambridge, Cambridge, United Kingdom; b Department of Psychiatry and Psychotherapy, Bethel Evangelisches Krankenhaus, Bielefeld, Germany; c MRC Unit for Lifelong Health and Ageing, University College London, London, United Kingdom; d College of Medicine and Veterinary Medicine, University of Edinburgh, Edinburgh, United Kingdom; e Department of Rehabilitation and Aged Care, Hospital Ancelle, Cremona, Italy; f Regenstrief Institute, Inc. and Indiana University Center for Aging Research, Indianapolis, Indiana; g Department of Psychiatry and Behavioral Sciences, Johns Hopkins University School of Medicine, Baltimore, Maryland; h Department of Psychiatry, Yale University School of Medicine, New Haven, Connecticut; i Centre for Cognitive Ageing and Cognitive Epidemiology, University of Edinburgh, Edinburgh, United Kingdom

**Keywords:** Delirium, dementia, epidemiology, systematic review

## Abstract

Delirium is a serious and common acute neuropsychiatric syndrome that is associated with short- and long-term adverse health outcomes. However, relatively little delirium research has been conducted in unselected populations. Epidemiologic research in such populations has the potential to resolve several questions of clinical significance in delirium. Part 1 of this article explores the importance of population selection, case-ascertainment, attrition, and confounding. Part 2 examines a specific question in delirium epidemiology: What is the relationship between delirium and trajectories of cognitive decline? This section assesses previous work through two systematic reviews and proposes a design for investigating delirium in the context of longitudinal cohort studies. Such a design requires robust links between community and hospital settings. Practical considerations for case-ascertainment in the hospital, as well as the necessary quality control of these programs, are outlined. We argue that attention to these factors is important if delirium research is to benefit fully from a population perspective.

Epidemiology is concerned with interrelationships between populations, exposures, and outcomes. The goal of the current article is to explore how the application of epidemiologic principles might provide opportunities for developments in delirium research. Epidemiology is able to address fundamental clinical questions in delirium:

• Who gets delirium? What are the risk factors that lead to its development? Who is predisposed? What are the precipitants?
• What is the natural history? How long might it last? What are the long-term outcomes? Are any adverse sequelae independent of the general consequences of systemic illness, trauma, surgery, or drug treatments?
• Is there an association with dementia? How strong is this association? Does delirium affect trajectories of cognitive decline?

Most of these questions have been addressed by studies in a range of settings. However, very little delirium research has been undertaken from a population-based perspective. This is essential if we hope to contextualize the many strands of investigation, otherwise limited by virtue of selected samples, within a common denominator.^1^

This article includes two parts: (1) a theoretical framework for epidemiologic research relevant to all older adults, namely: population selection, case-ascertainment, attrition (loss to follow-up), and confounding; and (2) a discussion on a critical question in delirium research: What is the relationship between delirium and trajectories of cognitive decline? Accordingly, it includes two systematic reviews: (1) the descriptive epidemiology of delirium in population-based studies; and (2) the impact of delirium on cognitive outcomes. These identify gaps, which lead to recommendations on how such an epidemiologic study of delirium and trajectories of cognitive decline might be practically achieved. We consider how to standardize and quality-control assessments of individuals with delirium. Thus, a range of specific clinical and organizational questions can be addressed.

## Part 1: Challenges in the Epidemiology of Psychiatric Syndromes

### Understanding the Provenance of Populations and Sample Selection

In considering the importance of defining a population, we are asking: Is the chosen population representative of the full spectrum of persons with delirium in that population? For example, if we are studying incidence of postoperative delirium in patients aged 70 years and older with urinary tract infections, are the individuals in the study representative of everyone with delirium or are there biases that arise because this is a relatively easy group to identify and recruit? How does the approach to sampling enable a valid capture of the chosen population? These are critical questions because the provenance of the sample population has the potential to systematically bias findings both in magnitude and direction.

The majority of studies in delirium have been undertaken in specific hospital settings and often among patients with particular medical or surgical conditions.^2^, ^3^ Together, these studies indicate that delirium is a common problem in inpatients and is associated with serious adverse outcomes, such as increased mortality, institutionalization, and dementia. However, there are three limitations to the inferences that can be drawn about delirium as a whole in the existing literature. First, one cannot assume that all persons with delirium from a given population will actually present to the particular hospital from which the respondents come. Second, once in the hospital, there is only retrospective information on a person's cognitive and functional status. This lack of reliable data on preadmission status makes it difficult to ascertain delirium (and pre-existing dementia) because the diagnosis requires determination of acute change in mental status. Third, the referral and selection bias inherent in hospital-based studies with particular subgroups of people with delirium leads to questionable generalizability or often conflicting findings across studies.

An example of the importance of working with an unselected population is evident from the findings of the Oxfordshire Community Stroke Project (OCSP)^4^, ^5^ and its successor, the Oxford Vascular Study (OXVASC).^6^ A working definition for population-based study might be: “a study where all subgroups of the population are sampled, regardless of disease or residential status.”^7^ These studies of stroke incidence made comprehensive efforts to ascertain all cases of transient ischemic attack (TIA) or stroke from a defined population registered at general practitioners' (GP) offices, where virtually all primary care in the United Kingdom is delivered. Each participating surgery (clinic) maintained close personal contact with the study, and collaborating GPs reported suspected cases to the study as soon as patients presented. If participants were not admitted to the hospital directly, they were assessed on the day of referral in a dedicated research clinic or at the participant's own home. All computerized diagnostic codes were reviewed, strengthened by record linkage systems between primary and secondary care. Hospital and emergency department presentations were reviewed daily, and all deaths out of hospital were identified via the coroner's office.

This strategy to include all cases from the general population resulted in great advances in understanding the prognosis and outcomes from TIA and stroke, precisely because it included the full range of persons with acute neurovascular events. In a systematic review of studies reporting the risk of early stroke after TIA, it is clear that population-based studies had much higher estimates of early recurrence (within 7 days) compared with those samples presenting solely to specialist stroke services (proportion (95% confidence interval [CI] recurring within 7 days in population-based studies versus specialist stroke services: 10.4% [8.1–12.6)] versus 0.9% [0.0–1.9], respectively).^8^ It is now clear that the relationship between TIA and early stroke can be predicted by using a clinical risk score.^9^, ^10^ These findings had a major impact in the planning of stroke services and in improving outcomes for patients.^11^

For delirium research, we need to consider how explicitly the population is defined. To understand how delirium relates to adverse cognitive outcomes, an optimal design would start with a broad, unselected denominator (i.e., a true population-based study) followed up with serial cognitive, mood, and functional assessments. This method would result in the identification of a comprehensive range of symptoms and severities, and would establish what happens, to whom, and when. Of course, ensuring that a study population is comprehensive in this way requires substantial effort, but there are gains of equal degree in terms of achieving results with external generalizability.

### Case Ascertainment in Research: Problems With Respect to Delirium

To reliably track states of health in populations, the definition of exposures and outcomes of interest must be standardized. For psychiatric syndromes, the reference-standard definition is necessarily a set of clinically agreed on descriptions of psychopathology rather than any objective measures. However, the possibility that biomarkers might eventually contribute to case-ascertainment is reviewed in the following discussion.

#### From definition to operationalization

There are some differences between the *International Classification of Diseases* (World Health Organization) and the *Diagnostic and Statistical Manual of Mental Disorders* (DSM) (American Psychiatric Association) definitions of delirium, and these have an impact on case-ascertainment.^12^, ^13^, ^14^ These definitions evolve with each revision and are therefore not stable over time. More problematic is that these clinical criteria have the potential to be interpreted differently by individual clinicians. For example, the threshold for impairment on cognitive testing in delirium may decrease with age, in line with a belief that some deficit is expected, and thus not abnormal, in older age.^15^

It is worth examining the precision of the *Diagnostic and Statistical Manual of Mental Disorders, Fourth Edition, Text Revision*, description, exploring the difficulty with using the definition for standardizing case-ascertainment in research. Although successive revisions are supposed to be based on epidemiologic field testing, only two studies were conducted in tertiary hospital samples (total n = 560) for *Diagnostic and Statistical Manual of Mental Disorders, Fourth Edition* (DSM-IV).^13^, ^16^

Deficits in attention have been recognized as a core diagnostic feature since publication of *Diagnostic and Statistical Manual of Mental Disorders, Third Edition Revised* (DSM-III-R) (Criterion A) (Box 1). It supplanted the previous description “clouding of consciousness” because the latter term was regarded as being too imprecise.^17^ However, it is not clear what should constitute a minimum threshold for attentional deficits in the diagnosis of delirium using DSM-III-R and above.^18^ Moreover, patients who present with a reduced level of consciousness in an acute setting are often not included in delirium studies if the severity of their impairments means that they cannot undergo cognitive testing. These two unresolved but crucial issues reflect the general scarcity of research on the neuropsychology of delirium.^19^

Box 1 **Diagnostic and Statistical Manual of Mental Disorders, Fourth Edition, Criteria for Delirium**

**Table:** 

A. Disturbance of consciousness (i.e., reduced clarity of awareness of the environment) with reduced ability to focus, sustain, or shift attention.
B. A change in cognition or the development of a perceptual disturbance that is not better accounted for by a pre-existing, established, or evolving dementia.
C. The disturbance develops over a short period of time (usually hours to days) and tends to fluctuate during the course of the day.
D. There is evidence from the history, physical examination, or laboratory findings that the disturbance is caused by the direct physiologic consequences of a general medical condition.

*Diagnostic and Statistical Manual of Mental Disorders, Fourth Edition, Text Revision,* also requires a change in cognition or perceptual disturbance (Criterion B). However, the extent to which delirium may have a differential effect on domains of cognition or perception is complex and not specified. Neuropsychiatric symptoms such as motor ^20^ or sleep–wake^21^ disturbance are frequently present but not specific for delirium. Affective symptoms, thought disorder, and perceptual disturbances are also recognized as part of Criterion B, and operationalizing these features would serve to maximize sensitivity of detection.

Criterion C states that symptoms should be acute (hours to days) and fluctuate over the course of the day. These features are highly specific to delirium. However, by their nature, they make ascertainment more difficult because a test score may vary over periods of hours or even minutes. Multiple assessments per day could increase detection of deficits as well as eliciting fluctuation but may be impractical. Currently, best practice is to use tools that attempt to capture relevant information (e.g., informant history, clinical case notes) in the period preceding the assessment.

Specifying that delirium is due to an underlying medical disorder fulfills Criterion D. However, it is unclear what should actually constitute “evidence” for cause and effect. For the vast majority of cases, acute medico-surgical events (e.g., urinary tract infection) and delirium are temporarily linked. However, because the pathophysiology of delirium remains elusive,^22^ level of evidence for etiologic links remains subject to scepticism. In addition, often multiple etiologies are demonstrable over the course of delirium^23^ but may be unidentifiable in ∼10%.^24^ It is not known if the precipitant influences the phenomenologic presentation.

#### Delirium and dementia

The boundaries for the delirium syndrome become more complex when considering co-morbid dementia. DSM separates the delirium and dementia definitions, but the problem of identifying one superimposed on the other remains. This is crucial because delirium can be missed, under an assumption that observed cognitive deficits are due to dementia. When delirium and dementia co-exist, the delirium symptoms (e.g., prominent inattention with fluctuating deficits) are thought to dominate the presentation over the impairments seen in dementia; this theory has been reviewed in detail elsewhere.^24^, ^25^, ^26^ However, much of the delirium fieldwork explicitly excluded persons with dementia; therefore, the resultant conceptualization overemphasizes features that are more likely to be reported in cognitively intact persons (e.g., psychotic symptoms). Conversely, delirium scales that include assessments of memory or other cognitive deficits known to be present in dementia (such as the Delirium Rating Scale–Revised-98)^27^ may be confounded by the presence of dementia. Moreover, some delirium assessment instruments have been validated in groups from which dementia patients were excluded. One consequence is that individuals perform poorly on the memory subscale because of dementia, regardless of whether delirium is also present. Currently, it is not known if delirium and dementia can be distinguished in a cross-sectional assessment on cognitive and phenomenologic grounds alone, but some studies suggest that this might be possible.^28^, ^29^, ^30^

The chief difficulty with operationalizing delirium is that the boundaries of the main constructs are not clearly defined. DSM does not specify duration, severity, minimum thresholds, or which symptoms should fluctuate over which time frames. However, empiric data suggest that each of these parameters may influence outcomes and therefore perhaps define prognostic groups (Table 1).^24^, ^26^ Further detailed population-based fieldwork involving increased use of standardized definitions and measurements with objective high reliability is essential if case definitions are to describe useful phenotypes. Despite these limitations, in the research setting, the aim is to operationalize these criteria so that case-ascertainment can be achieved in a consistent manner.

**Table 1:** Clinical Features in Delirium Not Currently Defined by DSM Criteria With a Theoretical Influence on Determining Prognostic Categories

	Effect on Prognosis	Comment	References
Motoric subtypes	Hypoactive delirium associated with higher mortality, especially with co-morbid dementia	Motoric assessment, including accelerometer-based measures have scope to inform prognostic categories	79, 80, 81
Duration	Minimum and maximum duration unclear	Delirium may evolve into dementia. Short-term versus persistent delirium proposed in DSM-5 (threshold not specified)	
Temporal fluctuations	Specifying short fluctuations (hours) favors identification of hyperactive over hypoactive subtype	Hypoactive delirium has poorer prognosis; therefore, any specification of temporal fluctuations should take this into account	
Severity	Clinical rating scales in existence (e.g., DRS-98, MDAS, Delirium Index). Higher scores associated with worse outcomes	Categories of severity might be incorporated into diagnostic criteria	82
Subsyndromal delirium	Higher mortality and worse cognitive outcomes compared to normal controls	Variably defined; represents a state between normality and full delirium syndrome. Current definition of delirium might perhaps be broadened to include milder deficits	83, 84

*Notes:* DRS-98: Delirium Rating Scale–Revised-98; DSM-5: fifth edition of the *Diagnostic and Statistical Manual of Mental Disorders*; MDAS: Memorial Delirium Assessment Scale.

#### Biomarkers and psychiatric syndromes

Biomarkers have been widely considered in dementia, for example, in the hope that a greater understanding of dementia pathophysiology might be able to contribute to case-ascertainment or even supplant the current clinical reference standard.^31^, ^32^, ^33^ There has been substantial progress in the field; for example, identifying amyloid burden in vivo^34^ and putative markers of neurodegeneration, among others.^35^, ^36^ However, such work has only ever been generalizable to the selected populations able to tolerate procedures such as positron emission tomography/magnetic resonance imaging or lumbar puncture.^1^

There is a real need for biomarker research to be validated within the context of a general population before they can be proposed as part of a new reference standard. Current plasma biomarker candidates such as apolipoprotein E, insulin-like growth factor-1, and S-100β for predicting delirium risk, prognosis, or severity have recently been reviewed.^37^ Other candidate biological correlates of delirium include electroencephalogram,^38^ neuroimaging,^39^ and markers in cerebrospinal fluid.^39^ It is clear that biomarkers in delirium are still in their infancy, but advances in our understanding of delirium pathophysiology may eventually help to refine case-ascertainment.

In conclusion, the optimal operationalization of DSM-IV for delirium would require: (1) a reliable and valid test of inattention; (2) reliable and valid assessments of cognition and neuropsychiatric symptoms; and (3) temporal nature of acute change captured by regular observation, with or without a contribution from informants. Ultimately, validation studies of biomarkers could be undertaken in unselected populations, serving to improve delirium knowledge at the clinical and population levels.

### Dropout in Studies of Older Adults: Accounting for Attrition

Loss to follow-up is common to all longitudinal studies of older persons. Reasons for loss of follow-up include dropout and death between interviews. This is also known as censoring, where individuals contribute to the observed period of follow-up but where loss to follow-up means that case status cannot then be ascertained. There is a clear effect on how accurately associations with outcomes can be made. Elaborating how these biases can be addressed is relevant for all follow-up studies of delirium.

It is important to explore possible reasons why outcome data may be missing. This involves considering whether the fact that data are missing might be associated with any other variables known (and unknown) in the study. Three characterizations of missing data mechanisms have been proposed: missing completely at random, missing at random (MAR), and missing not at random (MNAR) (Table 2).^40^ Data MNAR is the most likely mechanism to operate in longitudinal studies of aging, where the probability of an individual not being seen at a certain occasion depends on the individual's delirium status (unobserved) at that same occasion.

**Table 2:** Theoretical Mechanisms for Missing Data

	Definition	Example	Implications for Delirium Research
MCAR	Does not depend on observed or unobserved data	Lost data due to technical error such as miscalibration of MRI machine	Missing data are ignorable, but this is a rare situation
MAR	Depends on observed data	Unable to tolerate MRI sequences, predictable from knowledge of participant's cognition or ADL	Other parameters may explain the mechanism of missingess but not fully enough to provide unbiased estimates in analyses
MNAR	Depends on the value the outcome would have taken had it been observed	Attrition through death, driven by incident delirium or dementia that was not captured by the follow-up schedule	The most common mechanism of missing data in aging research. Requires specific and robust mechanisms for case-ascertainment, with statistical analyses to account for attrition

*Notes:* ADL: activities of daily living; MAR: missing at random; MCAR: missing completely at random; MNAR: missing not at random; MRI: magnetic resonance imaging.

Several approaches are available to analyze incomplete data. The simplest method consists of excluding cases with missing observations. This method, known as complete case analysis, is a very inefficient way of analyzing data and does not make use of all available information. Because data in longitudinal studies of older persons are unlikely to be missing completely at random, such an approach will bias the analysis in favor of better-performing participants. This illustrates why missing data cannot simply be ignored; the very fact that some data are missing is informative, and an appropriate analysis must be adopted.

Other ad hoc methods are based on the idea of “filling in” or imputing missing values to complete the data. Imputation has been proposed as a method of accounting for missing data on exposures (independent covariates) and outcomes (dependent variables). However, it should be noted that imputing outcomes is intrinsically problematic. This is because studies aim to determine a given outcome, and arguably, it would be unsatisfactory for this to be simulated. Methods include: last-observation-carried-forward imputation, regression mean imputation, and multiple imputation. For example, if a study examining the relationship between delirium (independent variable) and pressure sores (dependent variable) had data missing due to participants being too drowsy to participate in cognitive testing. Here, imputation could be used to account for these missing data points by estimating new values according to the pattern of loss in relation to other covariates in the model (e.g., illness severity).

If imputation for missing outcome data is to be avoided, other analytical approaches are recommended. If we are prepared to assume missing data are MAR, random-effects modeling is a statistical technique that produces robust estimates and uses all available data. However, if we believe that an MNAR mechanism might be a more reasonable assumption to make, then more sophisticated statistical techniques such as shared parameter models might be the most adequate method of analysis.^41^ The use of these techniques is not widespread in the literature on aging, but researchers who believe that a MAR assumption is not valid in their studies (because information on delirium “missingness” is not in the model [e.g., attrition due to higher delirium-specific mortality]) should consider applying these more refined analytical methods. Although these approaches have been important in dementia epidemiology, they have yet to be applied systematically to follow-up studies of delirium, which almost certainly underestimate the effect of dropout.^42^, ^43^, ^44^

### Accounting for Predisposing and Precipitating Factors: Residual Confounding?

Observational epidemiology seeks to identify associations between exposures (independent variables) and outcomes (dependent variables). Delirium can be regarded in both contexts. For example, delirium might be modeled as an exposure, with dementia as an outcome. Alternatively, sometimes delirium is considered the outcome, in which, for example, statin therapy is the exposure. These analyses should be undertaken with attention to the possibility of confounding.

The psychiatric formulation identifies two dimensions that need to be accounted for when considering prospective associations in delirium studies: precipitating and predisposing factors. Precipitating factors include measures of acute illness severity (which may include measures of intensity of surgery), and predisposing factors include cognitive impairment and frailty. There is an inverse relationship between these two dimensions: relatively minor illness precipitants may result in delirium given predisposing dementia.^45^ This is clinically intuitive and has long been supported by empiric data.^46^

Can we account for the effects of predisposing and precipitating factors so that we can assess the independent associations with delirium? In other words, is delirium directly responsible for the association in question or is it a marker for some more fundamental, less-measurable mechanism? This problem was recognized in a systematic review of outcomes after delirium in hospitalized patients, in which one of the inclusion criteria was that studies should have adjusted for co-morbid illness or illness severity.^3^

The review considered predisposing and precipitating factors together, and the individual studies operationalized these dimensions as follows:

• Predisposing factors: for example, presence of dementia or cognitive test score such as MMSE and the Informant Questionnaire on Cognitive Decline in the Elderly; Charlson co-morbidities index; functional measures such as activities of daily living.
• Precipitating factors: for example, acute physiology and chronic health evaluation II (APACHE II) score (Acute Physiology scale); physiologic or metabolic parameters: systolic blood pressure, C-reactive protein, urea, creatinine.
• Scales combining assessments of both factors: for example, the Burvill scale (a physician judgment–based scoring of several organ systems in which severity of acute and chronic conditions and their contribution to disability are assessed).

All studies made an attempt to adjust for predisposing factors, suggesting that it is easier to operationalize this dimension. To account for illness severity, many studies used APACHE II as a measure of both predisposing and precipitating factors, which has never been validated outside the intensive care unit or in older persons.^47^ The other approach to adjusting for illness severity was to use a marker of overall metabolic or physiologic derangement (e.g. C-reactive protein levels, elevated urea/creatinine ratio).

Very few prognostic models for mortality have been validated in individuals aged 50 years and older.^47^ Perhaps the most well established is the Charlson co-morbidities index,^48^ which provides a weighted score representing co-morbidities (and therefore chronic predisposing factors). One problem is that the weightings and the conditions were validated more than 20 years ago, and secular trends may therefore limit its validity. For example, a diagnosis of acquired immunodeficiency syndrome scores the same as metastatic disease, and peptic ulcer disease is weighted the same as congestive heart failure.

Overall, the question remains as to how to reliably detect and, where possible, quantify acute precipitating factors in delirium. Another approach from the acute internal medicine literature examines “early warning scores.” The best performing tool to date is the VitalPAC early warning score.^49^ This was devised to predict in-hospital mortality within 24 hours of acute admission and uses a weighted aggregate of seven parameters: pulse rate, respiratory rate, temperature, systolic blood pressure, oxygen saturation, inspired oxygen, and level of consciousness. The model was validated on 35,585 patient episodes, and the median age was 68 years. This approach has not been considered before in delirium but could be valuable. However, many of these indicators may not perform in the same way in older people,^50^ and measures of level of consciousness overlap with many symptoms of delirium.

In Part 1, we outlined a theoretical framework of epidemiologic principles. Attention to population selection, case-ascertainment, attrition, and confounding is vital if delirium is to be investigated in a valid and reliable way. Part 2 identifies gaps in the existing literature with regard to these concepts, proposing some recommendations for addressing a specific question in delirium epidemiology: the relationship between delirium and trajectories of cognitive decline.

## Part 2: Epidemiologic Approaches to Delirium and Trajectories of Cognitive Decline

This section focuses on the methodologic issues central to a key question in delirium research: what is its relationship with trajectories of cognitive decline? This question has wide-ranging biological, clinical, and public health implications. If delirium is robustly associated with trajectories of cognitive decline, then delirium prevention might plausibly have an impact on dementia prevention. Therefore, demonstrating an association with morbidity and economic costs, and quantifying these relationships within a population-based setting, presents a strong case for such epidemiologic research.

Before setting out how a study of this nature might be achieved, two systematic reviews are presented, one on population-based descriptive epidemiology and one on cognitive outcomes after delirium in prospective studies, which together provide an indication of the quantity and quality of previous work in this field. The first reviews population-based studies reporting the descriptive epidemiology of delirium (in the section entitled “Descriptive Epidemiology of Delirium in Population-Based Studies”). The second reviews cohort studies reporting the impact of delirium on trajectories of cognitive decline in which adverse cognitive sequelae can be reliably assessed with reference to pre-morbid cognitive assessments (in the section entitled “Delirium and Cognitive Trajectories”). It is proposed that these different methods could be eventually combined in a unifying population-based study.

The last part of this section suggests practical approaches to case-ascertainment in epidemiologic studies, including the quality control of this process.

### Systematic Reviews

#### Methods

Both reviews shared a common protocol, following the meta-analysis of observational studies in epidemiology guidelines.^51^

##### Eligibility criteria

*Descriptive epidemiology:* Cross-sectional (prevalence) or cohort (prevalence and incidence) studies reporting delirium measures were considered. Studies were required to define delirium according to a standardized classification system and be conducted in groups sampled from the whole population unrestricted by residential or health status.

*Cognitive trajectories:* All prospective studies reporting delirium and subsequent cognitive impairment were eligible for inclusion. The study design needed to compare cognitive function before delirium with impairments afterward. Premorbid cognitive function must have been assessed by use of a neuropsychological evaluation. Retrospective estimates of premorbid cognition (e.g., Informant Questionnaire on Cognitive Decline in the Elderly, AD8) were not regarded as sufficiently reliable. There was no requirement for the population to be sampled in an unselected manner.

##### Search strategy and data extraction

A systematic search of MEDLINE, Embase, and the Science Citation Index (all from 1980–December 31, 2012) was conducted (Box 2). Studies before 1980 were not considered because such work would have predated *Diagnostic and Statistical Manual of Mental Disorders, Third Edition*, and therefore would be unlikely to report standardized case-definitions. Comprehensive text word, Medical Subject Headings, and Emtree terms were used to find relevant studies. Bibliographies of included articles and other reviews were screened, including use of forward matching. Three authors (D.H.J.D., A.J.H., A.M.) independently screened articles for inclusion. Estimates of prevalence and effect sizes in relation to cognitive outcomes (and their standard errors) were extracted, along with any relevant clinical variables, specifically: age, gender, and education (where reported).

Box 2 **MEDLINE (Ovid SP) search strategy**

**Table:** 

Systematic review 1
1. exp Delirium/ep [Epidemiology]
2. delirium.mp or “acute confusion”.mp or “metabolic encephalopathy”.mp
3. community or population
4. prevalence or incidence
5. (#1 OR #2) AND #3 AND #4
Systematic review 2
1. exp Delirium/ep [Epidemiology]
2. delirium.mp or “acute confusion”.mp or “metabolic encephalopathy”.mp
3. (cogniti*) AND (trajector* or decline or impairment)
4. dementia
5. (#3 OR #4)
6. (prospective or cohort)
7. (#1 OR #2) AND #5 AND #6

##### Data analysis

Statistical analyses were performed by using Stata version 12.1 (Stata Corp, College Station, TX).

*Descriptive epidemiology:* The range of different populations identified across the studies justified use of random-effect models for pooling the estimates.^52^ 95% CIs were calculated, and statistical heterogeneity was assessed by using the τ2 statistic.

*Cognitive trajectories:* These results were summarized in a narrative manner.

### Descriptive Epidemiology of Delirium in Population-Based Studies

**Figure 1:**
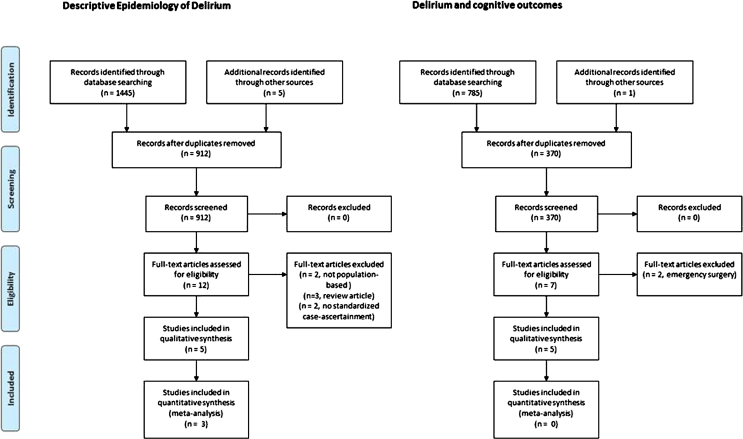
PRISMA flow diagram.

The identification, assessment, and selection of articles for inclusion is shown in the PRISMA flow diagram (Fig. 1). Three studies reported point-prevalence, and two reported period-prevalence (1 month and 3 years) of delirium. No population-based studies explicitly observing participants for incident delirium were identified. Characteristics of these studies are summarized in Table 3.

**Table 3:** Characteristics of Studies of Delirium Prevalence in the General Population

	Population and Setting	Design	Delirium Ascertainment
Point-prevalence			
East Baltimore Mental Health Survey^53^	Census blocks, random sample 18–64 years old and all residents age 65 years and older	NIMH DIS, with clinical assessment of random subsample (n = 398) and any others with positive DIS response (n = 412)	Standardized Psychiatric Examination and psychiatric assessment (DSM-III and ICD-9)
CSHA^55^	Random sample of all adults age 65 years and older clustered in five regions of Canada, over sampling adults age 75 years and older	Clinical examination of random subsample (n = 2,914) including all institutionalized adults and screen-positives for cognitive impairment (3MS <78/100)	DSM-III-R applied at consensus conference based on neuropsychiatric evaluation from nurse and physician. Delirium given as primary diagnosis if no underlying dementia diagnosis
Girona^54^	Door-to-door sampling of adults age 70 years and older (n = 1,581 eligible)	All screened participants (n = 1,460) with MMSE scores <24 (n = 335) and random sample with MMSE scores ≥24 (n = 314)	Neurologist- and psychologist-administered CAMDEX
Period-prevalence			
GERDA^56^	All women aged 90 years and older, 50% of those aged 85–89 years	All participants (n = 503) examined by using MMSE and OBS scale	DSM-IV applied based on study information, informant/caregiver interviews, medical records (1-month period)
Vantaa 85+^57^	Recruitment of all adults resident in Vantaa aged 85 years and older (n = 601 eligible)	All participants assessed with informant, with clinical, cognitive, and functional examinations	History of delirium established by retrospective interview of participant and informant with reference to medical case notes

*Notes:* 3MS: Modified Mini–Mental State Examination; CAMDEX: Cambridge Mental Disorders of the Elderly Examination; CSHA: Canadian Study of Health and Aging; DIS: Diagnostic Interview Schedule; DSM-III: *Diagnostic and Statistical Manual of Mental Disorders, Third Edition*; DSM-III-R: *Diagnostic and Statistical Manual of Mental Disorders, Third Edition Revised*; DSM-IV: *Diagnostic and Statistical Manual of Mental Disorders, Fourth Edition*; GERDA: Gerontological Research Database study; ICD-9: *International Classification of Diseases, Ninth Revision*; MMSE: Mini–Mental State Examination; NIMH: National Institute of Mental Health; OBS: organic brain syndrome scale.

#### Point-prevalence studies

All studies reporting point-prevalence used a basic screening measure, with more detailed characterization of screen-positive participants and a random subsample of screen-negative participants. The East Baltimore Survey ^53^ identified 6 cases of prevalent delirium, giving an age-specific prevalence of 10.9 (95% CI: 0.0–22.5) per 1,000 persons aged 55 years and older. It is not clear if any of these cases had co-existent dementia. A door-to-door survey of 1,460 individuals aged 70 years and older yielded 14 cases of delirium (prevalence = 9.6 [95% CI: 4.4–14.9] per 1,000 persons), 12 of whom also had dementia. The prevalence of delirium in persons with dementia was much higher: 79.5 (95% CI: 35–126) per 1,000 persons.^54^ The Canadian Study of Health and Ageing applied DSM-III-R diagnoses at consensus meetings after two independent neuropsychological evaluations.^55^ Diagnoses of delirium and dementia were considered mutually exclusive. The 21 delirium cases identified represented a point-prevalence of 6.3 (95% CI: 4.1–9.6) per 1,000 persons.

#### Period-prevalence studies

The Gerontological Regional Database (GERDA) study sampled women aged 85 years and older, and diagnoses pertaining to delirium in the previous month were decided by a geriatrician with access to all study neuropsychological evaluations, informant, and caregiver interviews and medical records, based on DSM-IV criteria.^56^ The 1-month period prevalence for delirium was 272 (95% CI: 235–312) per 1,000 persons. Delirium prevalence was strongly associated with age (85–89 years, 19%; 90–94 years, 24%; 95 years and older, 39%) and dementia (odds ratio [OR]: 5.8 [95% CI: 3.5–9.5] for clinical Alzheimer disease).

The Vantaa 85+ study ascertained delirium in the 3 years between baseline interview and follow-up by assessing participants, along with their informant(s), for a history of any episodes of delirium based on an operationalization of the DSM-III-R criteria.^57^ The reported history was corroborated with hospital case notes that were available at the time of assessment. Delirium was reported to have occurred in 100 (95% CI: 66–150) per 1,000 persons surviving to first follow-up.

**Figure 2:**
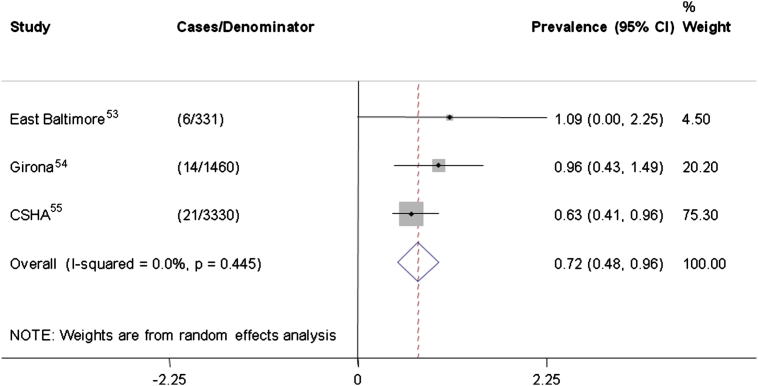
Forest plot and meta-analysis of point-prevalence studies. Estimates of delirium point-prevalence in population-based studies are shown, along with a pooled estimate. Numbers refer to age-specific prevalence per 1,000 persons, along with 95% confidence intervals (CISs). CSHA = Canadian Study of Health and Aging.

The estimates of point-prevalence are summarized in Figure 2. There is a consistent finding that population prevalence of delirium is relatively low, with an overall point-prevalence estimated at 7.2 (95% CI: 4.8–9.6) per 1,000 persons in the group aged 55 years and older.

#### Comment

Because the Canadian Study of Health and Ageing is the largest study and did not include delirium in persons with a dementia diagnosis, the pooled prevalence is also likely to be an underestimate. In addition, it is likely that intercurrent illness and/or delirium reduces response rates in epidemiologic surveys; the detected prevalence may therefore be very low by design. The period-prevalence seems to be higher in 1-month estimates compared with 3-year estimates (27% versus 10%) in a similarly aged population. This is likely to be driven by the association between delirium and mortality, and less delirium is thus observed in the 3-year survivors.

### Delirium and Cognitive Trajectories

Three reports from two cohort studies were identified. In addition, adverse outcomes in two studies of elective surgery patients were reported. The characteristics of these studies are described in Table 4.

**Table 4:** Characteristics of Studies of Delirium and Cognitive Outcomes

	Population	Exposure	Outcome	Comments
Cohort studies				
MADRC cohort^59^, ^60^	Memory clinic patients	Retrospective diagnosis of delirium from case notes based on CAM	Worsening on Blessed Information-Memory-Concentration score	Only considered persons with previous cognitive impairment
Vantaa 85+^58^	Community-based with generalized sampling frame	Participant and informant interview with access to medical records, based on DSM-III-R	Dementia, trajectory of MMSE change	True population-based sample. Delirium ascertainment retrospective
Elective surgical studies				
Bickel et al.^61^	Elective hip surgery patients aged 60 years and older	CAM-defined delirium	Cognitive impairment and/or dementia	Most follow-up assessments conducted by telephone interview
Saczyinski et al.^62^	CABG or valve surgery	CAM-defined delirium	Trajectory of MMSE change	

*Notes:* CABG: coronary artery bypass graft; CAM: Confusion Assessment Method; DSM-III-R: *Diagnostic and Statistical Manual of Mental Disorders, Third Edition Revised*; MADRC: Massachusetts Alzheimer's Disease Research Center; MMSE: Mini–Mental State Examination.

#### Cohort studies

In Vantaa 85+, a history of delirium was associated with incident dementia at follow-up (OR: 8.7 [95% CI: 2.1–35]).^58^ Delirium was also associated with worsening dementia severity (OR: 3.1 [95% CI: 1.5–6.3]) and with loss of 1.0 more MMSE point per year (95% CI: 0.11–1.89) than those with no history of delirium. In a cohort of memory clinic patients with dementia, delirium was associated with an additional decline of 2.4 points on the Information-Memory-Concentration scale over 6 months compared with those with no delirium.^59^ These differences were consistently observed over 5 years posthospitalization.^60^ Both of these studies relied on the diagnosis of delirium through retrospective review of medical records.

#### Elective surgery patients

In 200 patients undergoing hip surgery, postoperative delirium was associated with new cognitive impairment or dementia at 3 years, even after adjustment for preoperative MMSE in the delirium group (OR: 41 [95% CI: 4.3–396]).^61^ In a population of cardiac surgery patients, those with delirium had significantly lower postoperative MMSE scores but also slower recoveries, with worse scores at 1 and 6 months after surgery.^62^

#### Comment

These studies link baseline cognition, delirium, and worsened cognitive function. The two methodologic designs identified (cohort studies and studies in elective surgery patients) have complementary strengths and weaknesses. Studies in elective surgery patients had a better opportunity to describe the index delirium but in a more restricted sample and usually with less information about premorbid function. Conversely, cohort studies offer the possibility of repeated cognitive measures, before and after incident delirium. Moreover, cohort studies may be sampled from the general population, as in the case of Vantaa 85+. The disadvantage is the difficulty in prospectively capturing and characterizing the delirium episode. In both cohort studies identified, the delirium ascertainment relied on interview after the event and/or retrospective examination of case notes. Although these methods can be validated in terms of their diagnostic accuracy,^63^ they likely underestimate hypoactive forms of delirium, as well as those who do not present to a hospital. Nonetheless, these results are consistent with other studies that have considered critical illness as a proxy for delirium (and vice versa).^64^, ^65^, ^66^

### Delirium and Cognitive Decline: Recommendations for Epidemiologic Study Designs

#### Population

The conclusions from both systematic reviews indicate that there are very few population-based studies assessing delirium prevalence. However, it is probable that point-prevalence of delirium in the community is low. Nonetheless, the value of these studies is that they describe an approach to characterizing a base population, with the possibility of enriching it with groups likely to eventually yield more incident delirium cases (e.g., older subjects, persons with pre-existing cognitive impairment). Therefore, although the point-prevalence at any given moment may be low, in persons aged 85 years and older, the 1-month period-prevalence may be as high as 25%. More intensive follow-up of higher risk subsamples, randomly selected to maintain external generalizability, has been successfully used in a number of dementia studies and could be usefully considered here.^67^, ^68^

Essential to the recruitment of representative populations, issues surrounding capacity and consent in delirium studies must be addressed. The ethical framework for approaching this topic has been reviewed and highlights the need to protect vulnerable participants while also asserting the equal moral status for persons with delirium to have their condition researched in a valid way.^69^ Other studies have also demonstrated that methods used to assess capacity, including individuals with fluctuating capacity, had an effect on the research conclusions, depending on whether persons were included or excluded according to capacity status.^70^ In some circumstances, the use of proxy consent, especially for low-risk studies, may be a practical option.

#### Case-ascertainment

From the second systematic review, the next steps would be to establish a system whereby acute changes in mental status can be identified (e.g., via primary care practitioners). As in the OXVASC study (section entitled “Understanding the Provenance of Populations and Sample Selection”),^6^ this requires excellent links between hospital and community services. Use of GPs to notify study personnel of acute changes is likely to need dedicated resources to be effective. A brief screening instrument would be the first step for case-ascertainment. It is not known if delirium can be optimally diagnosed, investigated, and treated in the community, and the study should be able to determine the need for hospitalization.

**Figure 3:**
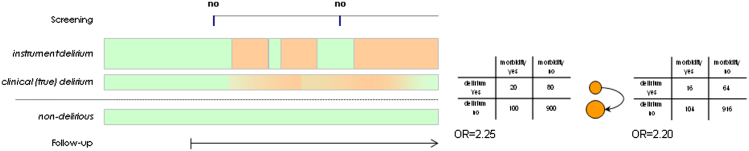
The effect of misclassification in delirium studies. Delirium is represented by peach shading in cases; green color indicates no delirium in cases and controls. The contingency tables demonstrate the effect of false-negative findings when assessments are not made with enough frequency. OR = odds ratio.

Once in secondary care, longitudinal delirium assessments must try to account for temporal fluctuations. Information on delirium severity and duration in relation to long-term outcomes would be an important and new finding in the general population. The assessment of candidate biomarkers could be incorporated both at this stage and earlier as an assessment of delirium vulnerability. The optimum examination schedule will be based on resources and patient tolerability. They may range from several (shorter) assessments several times daily or in other settings (e.g., in long-term care, twice-weekly assessments may be sufficient).^71^ In addition, the frequency of assessments minimizes the risk of misclassification bias. Figure 3 shows how this method increases the risk of erroneously accepting the null hypothesis through loss of statistical power. Other practical issues surrounding case-ascertainment is outlined in the next section.

#### Attrition and missing data

Procedures for determining outcomes need to be reliable, using data from multiple, overlapping sources. Missing data are to some degree unavoidable, and analyses must account for these with appropriate estimations of standard error. The random-effects models used in the cohort studies identified in the systematic review are generally flexible in this regard. However, missing data may well arise when competing outcomes are at play; for example, when dementia or death might follow delirium. Here, data on postdelirium cognition are “missing” because of intervening death between resolution of the delirium and the next follow-up in the cohort study. Techniques such as multistate or shared parameter models can be considered.

#### Residual confounding

Within the assessments for delirium and serial cognition function, other clinical factors need to be accounted for. Measurement of predisposing factors (e.g., age, gender, education, functional frailty) must be embedded in the assessment schedule and standardized with the same degree of precision as the delirium and cognitive variables. Illness severity may be more complex to capture, but basic physiologic parameters (such as those that comprise early warning score systems) have the advantage of being brief, reproducible, noninvasive, and repeatable. Repeatability is an important dimension because these measures of physiologic disturbance can then be tracked alongside fluctuations in delirium state.

### Standardization in Delirium Research: Best Practice for Future Research

#### Approaching case-ascertainment

There are several different approaches to delirium ascertainment in use. These include direct application of DSM-IV criteria, but rating scales are often used.^72^, ^73^ A recent review found 24 scales in the literature.^74^ These scales vary considerably in their complexity and in the procedures underpinning scoring each item. Some scales have a small number of binary items (e.g., the Confusion Assessment Method [CAM]), and others have several items with three or more severity gradations (e.g., Delirium Rating Scale–Revised-98). Most items in delirium scales are concerned with recording cognitive and neuropsychiatric features according to observations of the patient during the interview and/or a variable period preceding the interview. Features may be gleaned through discussion with informants or through case records.

Determining the presence or absence of these features is largely a clinical and subjective judgment. Some items on scales involve cognitive testing; these are more objective, but variations in how tests are administered and scored can reduce interrater reliability. In some studies, the authors have used a package of measures, which then inform scoring on scales, in an effort to standardize the mental status assessment preceding this scoring.^75^

Clearly, no consensus exists over what instruments should be used to capture delirium or subsyndromal delirium, or other forms of acute mental status deterioration occurring with illness, injury, or drug intoxication. Addressing this issue is an important priority in delirium research. There are no clear solutions, but it would seem reasonable to suggest the following, at least with respect to research studies. First, cognitive impairments are at the heart of delirium, and measurement of cognitive impairments by using objective tests provides meaningful data. Tests of attention, particularly those known to be greatly affected in delirium but not in dementia, have face validity for this purpose.^28^ Cognitive data could be reported along with binary delirium present or absent classifications, thus providing useful information on cognitive state that is independent of the particular delirium assessment being used. Second, rating scales that are anchored with graded behavioral descriptions likely have greater reliability than those asking for binary classifications. Third, in the absence of any consensus over the meaning of severe changes in level of alertness, but making the conservative assumption that these changes are not likely to be independent of delirium, more precise grading of level of alertness as part of the assessment would likely add useful information. This information could also be reported independently of the delirium classification. Such a package of measures would take time to administer, with associated additional costs, and empiric evaluation of the meaning of these additional data with respect to outcomes would be required before standard implementation is recommended.

#### Quality control in clinical studies: training protocols for standardized case-ascertainment

Because delirium is a clinical diagnosis, case-ascertainment by a psychiatrist, neurologist, geriatrician, or related specialist has been used as the reference standard in much of the assessment literature to date. Physician expert case-ascertainment compared with trained research nurses using a structured assessment protocol suggests that similar, if not improved, sensitivity and specificity can be attained.^76^ Large-scale studies linked to a community population would likely engage nonclinical personnel for the assessment of delirium. Currently, there is only one structured interview available, the Delirium Symptom Interview (DSI), with a sensitivity of 0.90 and a specificity of 0.80 compared with psychiatrists' and neurologists' assessment.^77^ Use of the DSI along with the CAM diagnostic algorithm, after performing standardized testing of cognition with instruments with the MMSE, Digit Span, and the Memorial Delirium Assessment Scale, has been reported to be reliable for nonclinicians as part of a standardized delirium assessment.^75^

The standardized assessment based on DSI was recently used in the multisite delirium prevention ancillary study to the FOCUS Hip Fracture Transfusion Trial.^78^ The ongoing Dexlirium study (ClinicalTrials.gov identifier NCT00561678), another multisite, randomized postsurgical delirium prevention trial, uses a similar standardized assessment protocol. One major drawback to the standardized assessment is its lengthy duration. In the FOCUS-Cognition and Dexlirium studies, the assessment can take up to 40 minutes. Also, despite the detailed nature of the standardized protocol, the eventual ascertainment of delirium status (e.g., based on the CAM algorithm) still requires a degree of clinical decision making. A reliable and valid but shorter standardized protocol that reduces this subjectivity for nonclinical personnel would be very useful for an epidemiologic study of delirium.

All personnel require ongoing training and quality-control efforts for consistency and interrater reliability in delirium assessment. A training protocol starts with instruction in basic aspects of delirium assessment and detailed review of the study instruments. A certification process will foster competency in the delirium assessor. The FOCUS-Cognition and Dexlirium studies used Web-based training that demonstrated conduct of the standardized assessment on a model patient, and tested the reliability of the trainee by asking them to rate a case based on a videotaped interaction between a model patient and the assessor. Validity of the trainee's assessment is further enhanced by an in-person training protocol in which the trainee undertakes the assessment under the supervision of a study physician. Because changes in study personnel occur frequently, quality-control efforts, such as co-rating in-person, videotaped interviews, or sequential examinations by different personnel of the same patient on the same day, are required throughout the duration of the study to demonstrate reliability of the delirium assessments. Although it is preferable to review every delirium assessment in a structured way by using all available evidence with a consensus panel, this might not be feasible in large epidemiologic studies. The Dexlirium and FOCUS-Cognition studies used a weekly or monthly teleconference throughout the duration of each study to discuss select delirium cases to improve the competency of the assessors and to clarify any issues related to assessment.

## Conclusions

It is clear that standardization is a complex issue in psychiatric epidemiology. At present, only clinical criteria can be used to define cases, although biomarkers may be promising if validated in unselected populations. One general consequence of not identifying delirium accurately is misclassification bias, leading to a reduction in the observed effect size. Thus, insufficient attention to standardizing case-ascertainment leads to loss of power in clinical studies.

It seems that many questions of direct clinical relevance to the understanding and management of delirium could be addressed by a convincingly designed observational study. Starting with a cognitively characterized, unselected base population, tracking individuals longitudinally in and out of hospital settings, is essential. Case-ascertainment would benefit from a more standardized application, perhaps including a battery of objective tests along with conventional subjective assessments, in consensus conferences and/or algorithmic operationalization. Fluctuating symptoms are a core feature of delirium, and this will not be reliably captured without specific attention on how this contributes to case-ascertainment. These efforts will be rewarded by generating methodologically rigorous clinical data applicable to the broad generality of patients with delirium.
